# Inferred retinal sensitivity in recessive Stargardt disease using machine learning

**DOI:** 10.1038/s41598-020-80766-4

**Published:** 2021-01-14

**Authors:** Philipp L. Müller, Alexandru Odainic, Tim Treis, Philipp Herrmann, Adnan Tufail, Frank G. Holz, Maximilian Pfau

**Affiliations:** 1 Department of Ophthalmology, University of Bonn, Ernst-Abbe-Str. 2, 53127 Bonn, Germany; 2 Center for Rare Diseases, University of Bonn, Bonn, Germany; 3 Moorfields Eye Hospital NHS Foundation Trust, London, UK; 4 Institute of Ophthalmology, University College London, London, UK; 5 BioQuant, University of Heidelberg, Heidelberg, Germany; 6 Department of Biomedical Data Science, Stanford University, Stanford, USA

**Keywords:** Retinal diseases, Hereditary eye disease, Biomarkers

## Abstract

Spatially-resolved retinal function can be measured by psychophysical testing like fundus-controlled perimetry (FCP or ‘microperimetry’). It may serve as a performance outcome measure in emerging interventional clinical trials for macular diseases as requested by regulatory agencies. As FCP constitute laborious examinations, we have evaluated a machine-learning-based approach to predict spatially-resolved retinal function (’inferred sensitivity’) based on microstructural imaging (obtained by spectral domain optical coherence tomography) and patient data in recessive Stargardt disease. Using nested cross-validation, prediction accuracies of (mean absolute error, MAE [95% CI]) 4.74 dB [4.48–4.99] were achieved. After additional inclusion of limited FCP data, the latter reached 3.89 dB [3.67–4.10] comparable to the test–retest MAE estimate of 3.51 dB [3.11–3.91]. Analysis of the permutation importance revealed, that the IS&OS and RPE thickness were the most important features for the prediction of retinal sensitivity. ’Inferred sensitivity’, herein, enables to accurately estimate differential effects of retinal microstructure on spatially-resolved function in Stargardt disease, and might be used as quasi-functional surrogate marker for a refined and time-efficient investigation of possible functionally relevant treatment effects or disease progression.

## Introduction

Recessive Stargardt disease (STGD1, Online Mendelian Inheritance in Man #248200), caused by biallelic mutations in the *ATP-binding cassette sub-family A member 4 (ABCA4*, Online Mendelian Inheritance in Man #601691) gene, is one of the main causes for inherited retinal degeneration and loss of vision in early life^1,2^. It leads to excessive accumulation of lipofuscin in the lysosomal compartment of postmitotic retinal pigment epithelium (RPE) that has been shown to have toxic effects on the RPE cells and photoreceptors^3,4^. It is clinically characterized by alterations at the posterior pole that can be visualized with digital imaging technologies as patterns of increased and decreased fundus autofluorescence (AF) on a background of increased AF intensity as well as thinning of retinal layers in the optical coherence tomography (OCT)^5–12^.

While sophisticated analyses of disease stages and progression based on morphologic changes have been proposed^8,9,13,14^, regulatory agencies have previously stated their preference for performance/functional outcome measures^15^. Best-corrected visual acuity (BCVA) is a practical, but not ideal marker of function due to its slow rate of change over time, individual variability, phenomena such as foveal sparing, and limited spatial representation restricted to the preferred retinal locus^16,17^. In this context, fundus-controlled perimetry (FCP, also termed microperimetry) is an established psychophysical assessment allowing for spatially-resolved measures of retinal sensitivity at different predefined retinal locations while compensating for fixation instability^18,19^. However, FCP requires dedicated equipment and is time-consuming. Due to limited examination time, every FCP test is a trade-off between the size of the test-field, spatial-accuracy (i.e., location and number of test-points) and threshold-accuracy (i.e., step size of the staircase strategy and number of reversals). The examination time is crucial concerning subjects’ fatigue and compliance (e.g. false-positive and false-negative responses).

OCT allows for axially resolved imaging of the retina, has been extensively investigated for retinal diseases, offers validated biomarker such as central retinal thickness in exudative maculopathies including neovascular age-related macular degeneration (AMD), and is widely available^20^. The lateral (or *en-face*) resolution of current OCT devices (up to 5.7 µm/pixel for the Spectralis OCT 2 device, Heidelberg Engineering, Germany) is more than one log unit higher compared to the typically used Goldmann III (128 µm diameter) stimulus in FCP testing. The preparation and acquisition time is short (i.e., no pupil dilation needed), and the imaging does not require long periods of subject engagement and alertness^21^. Recently, the possibility to predict FCP results based on structural OCT data (also termed ‘inferred sensitivity’) using artificial intelligence algorithms, including machine learning techniques like random forest regression, was described for AMD^22,23^. In the view of upcoming therapeutic trials and the attempt to keep study protocols slender while achieving maximal validity and power^24^, the prediction of reliable functional outcome measures based on fast and routine retinal imaging might be a reasonable solution.

In this longitudinal, natural history study of patients with STGD1, we (1) therefore investigated the accuracy of machine learning models to predict retinal function based on structural imaging data (‘inferred sensitivity’), (2) estimated the effect of measurement error and patient reliability on the modeling process, (3) assessed the relative importance of retinal biomarkers for the prediction, and (4) examined the ability to detect change over time.

## Results

### Demographic characteristics

**Figure 1:**
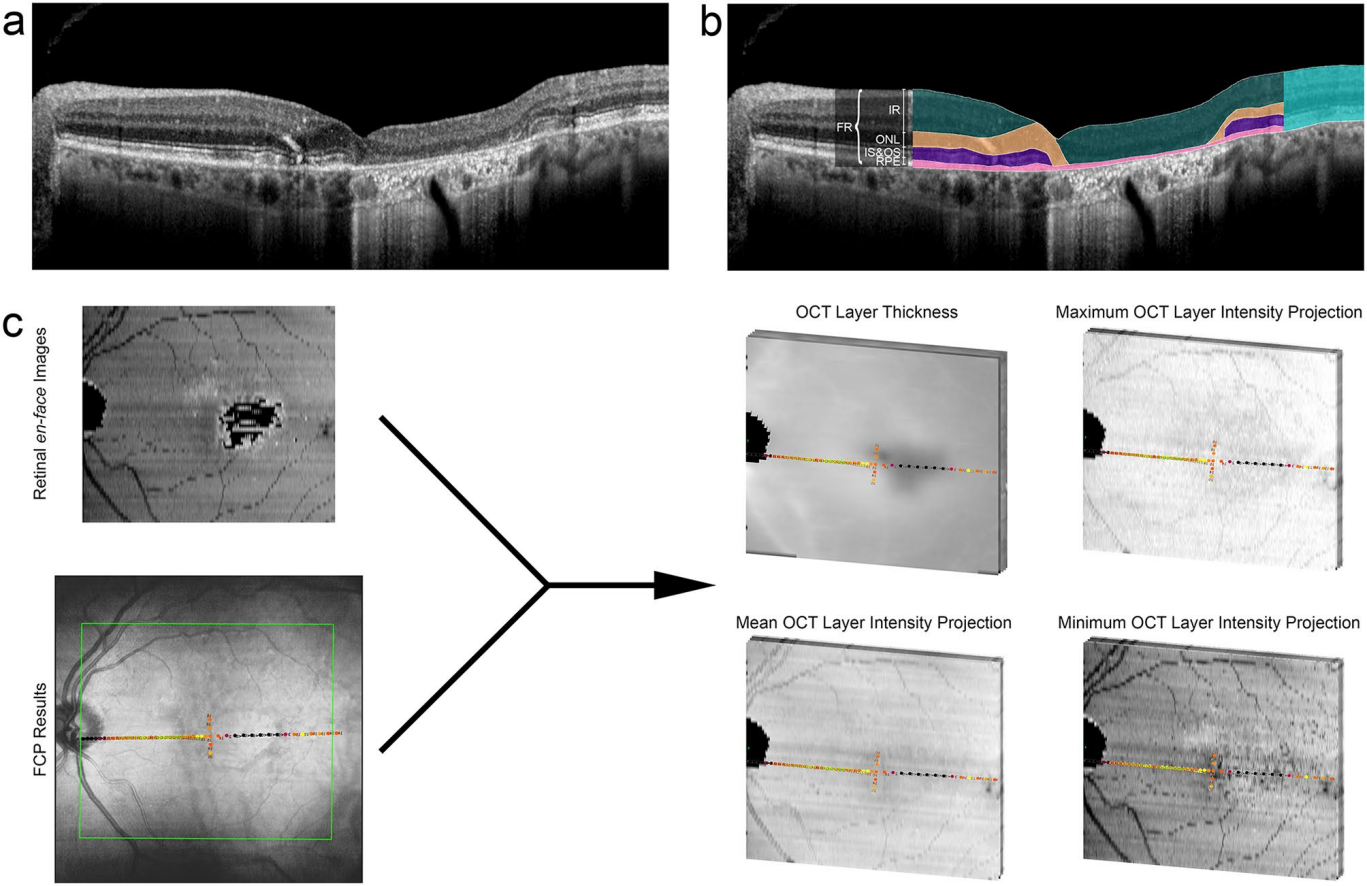
Image segmentation and multimodal registration. (**a**,**b**) The spectral-domain optical coherence tomography (OCT) volume scan was semi-automatically annotated. The herein used segmentations for the full retina (FR, bright blue overlay), inner retina (IR, green overlay), outer nuclear layer (ONL, yellow overlay), photoreceptor inner and outer segments (IS&OS, purple overlay), retinal pigment epithelium (RPE, pink overlay) are highlighted. (**c**) The fundus-controlled perimetry (FCP) results (color coded dots on infrared reflectance image) were registered to the *en-face* thickness and projection maps of the five OCT layers. The green rectanlge on the FCP results indicates the location of the respective OCT volume scan. For each stimulus location, the mean thickness as well as mean, maximal and minimal projection intensity were extracted for each layer.

A total of 267 eyes from 134 patients with STGD1 with a median (IQR) age of 37.1 years (22.0, 50.2) at baseline and 87 eyes from 54 controls (36 female, 18 male) with a median age of 41.0 years (25.7, 53.2) were included (Table 1). For all following analyses, patient data was standardized by normal data in consideration of the spatial differences in retinal sensitivity as well as OCT layer thicknesses and reflectivity intensities (cf. “Methods” section and Fig. 1). Accordingly, only patient data was used to derive the estimates for the prediction accuracies to obtain most conservative estimates.

**Table 1:** Patient baseline characteristics.

	Overall (baseline) cohort	Cohort with test–retest examinations	Cohort with longitudinal follow-up
**Patient-level data**			
Patients (n)	134 patients	92 patients	52 patients
Sex (female/male)	85/49	59/33	30/22
Age at first examination (median [IQR])	37.1 years (22.0, 50.2)	37.1 years (21.4, 49.1)	34.0 years (21.1, 44.2)
Age of onset categories (n)			
Early-onset	32 patients	22 patients	14 patients
Intermediate-onset	77 patients	56 patients	29 patients
Late-onset	25 patients	14 patients	9 patients
Electrophysiological subtypes			
ERG group 1	62 patients	47 patients	25 patients
ERG group 2	53 patients	33 patients	23 patients
ERG group 3	19 patients	12 patients	4 patients
Follow-up time (median [IQR])	n/a	n/a	2.16 years (1.21, 3.10)
Follow-up visits (median [IQR])	n/a	n/a	1 (1, 2)
**Eye-level data**^**a**^			
Eyes without foveal sparing (n)	175	80	62
Eyes with foveal sparing (n)	92	40	42
BCVA (median [IQR])	0.9 LogMAR (0.2, 1.0)	0.9 LogMAR (0.2, 1.0)	0.7 LogMAR (0.2, 1.0)
Fixation stability in terms of the 95% bivariate contour ellipse (median [IQR])	1.48 log_10_(deg^2^) (0.98, 1.75)	1.54 log_10_(deg^2^) (1.08, 1.70)	1.37 log_10_(deg^2^) (0.88, 1.68)

*BCVA* best-corrected visual acuity, *ERG* electroretinogram, *IQR* interquartile range.

^a^One eye at baseline was excluded due to prior retinal detachment.

In terms of age of onset subgroups, 32 patients were affected by early-onset STGD1 (≤ 10 years), 77 patients by intermediate-onset STGD1 (10 < age < 45 years) and 25 by late-onset STGD1 (≥ 45 years). At baseline, 62 patients were assigned to full-field electroretinogram (ERG) group 1, 53 patients to ERG group 2, and 19 patients to ERG group 3 (cf. “Methods” section). Longitudinal follow-up data was available for 52 STGD1 patients with a median review period of 2.16 years (1.21, 3.10) corresponding to a median of 1 (1, 2) follow-up visits. Further, follow-up data was available for 14 of the control subjects with a median review period of 1.64 years (0.99, 3.17) corresponding to a median of 1 (1, 1) follow-up visit.

### Accuracy of light sensitivity predictions in patients with STGD1

**Figure 2:**
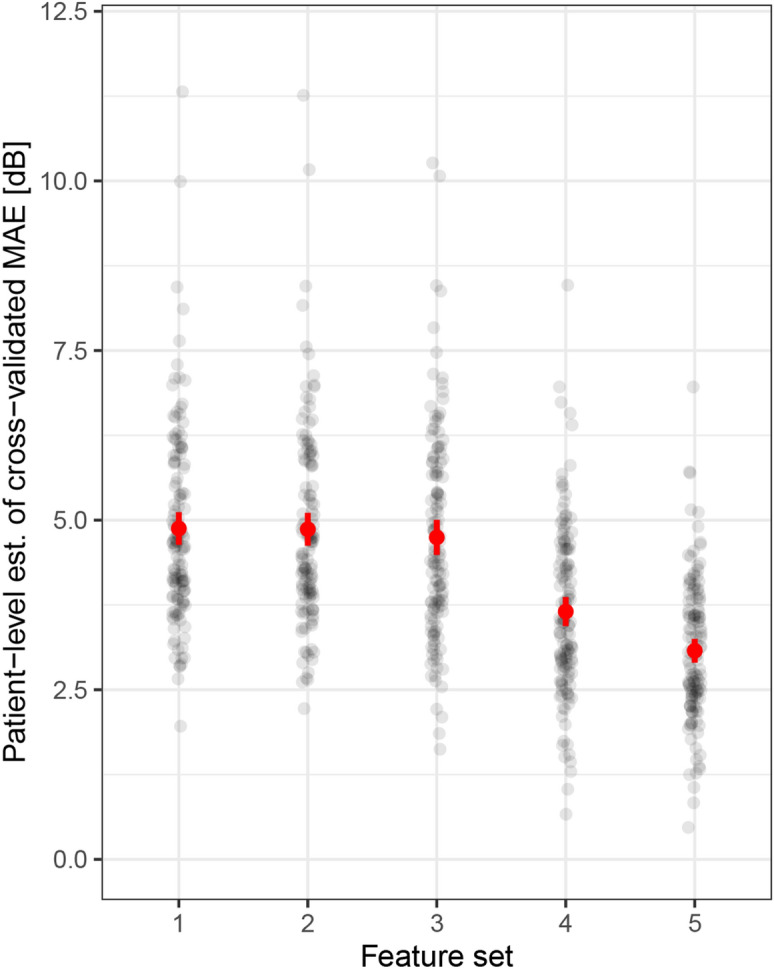
Patient-wise prediction accuracy. The figure shows the patient-level estimates (est.) for the mean absolute errors (MAE) between the predictions and observations in dependence of the five feature-sets. The red dot and error-bar indicate the mixed-model estimate for the MAE and the 95% confidence interval. To avoid overplotting, we used semi-transparent points. By adding additional patient specific features (i.e., feature-sets 4 and 5), the prediction accuracy could be markedly improved. *dB* decibels.

The cross-validated mean absolute errors (MAE [95% CI]) values obtained through the outer resampling of the nested cross-validation (i.e., without optimization bias, cf. “Methods” section, Supplementary Fig. S1) were 4.86 dB [4.62–5.09] for imaging data only (feature-set 1), 4.85 dB [4.61–5.08] with addition of patient reliability indices (feature-set 2), and 4.74 dB [4.48–4.99] with further addition of functional and demographic patient characteristics (feature-set 3, Fig. 2). All of these were markedly better compared to the representative null model (MAE of 10.80 dB [10.52–11.08]).

Analysis of the prediction error in dependence of the ETDRS subfields revealed, that the predictions errors were slightly higher for the central and inner ETDRS subfields as compared to the outer ETDRS subfields and peripheral retinal (Table 2).

**Table 2:** Mean absolute errors (MAE [in dB]) between retinal sensitivity predictions and observation.

Location	Feature-set 1		Feature-set 2		Feature-set 3		Feature-set 4		Feature-set 5	
	Estimates	CI	Estimates	CI	Estimates	CI	Estimates	CI	Estimates	CI
Overall	4.86	4.62–5.09	4.85	4.61–5.08	4.74	4.48–4.99	4.11	3.88–4.35	3.89	3.67–4.10
Nasal periphery	4.55	4.12–4.98	4.56	4.13–4.98	4.6	4.17–5.03	3.72	3.30–4.14	3.71	3.31–4.10
Nasal out. SF	4.7	4.45–4.95	4.66	4.41–4.92	4.58	4.31–4.85	3.89	3.63–4.15	3.69	3.45–3.92
Nasal in. SF	5.34	5.07–5.60	5.33	5.07–5.60	5.19	4.91–5.47	4.63	4.37–4.90	4.30	4.04–4.55
Central SF	5.37	5.09–5.65	5.38	5.10–5.65	5.08	4.79–5.37	4.53	4.25–4.81	4.42	4.16–4.69
Temporal in. SF	4.88	4.59–5.17	4.88	4.59–5.17	4.89	4.59–5.20	4.26	3.96–4.57	4.07	3.76–4.39
Temporal out. SF	4.34	4.06–4.62	4.34	4.06–4.61	4.27	3.98–4.57	3.58	3.28–3.89	3.29	2.97–3.60
Temporal periphery	4.37	4.08–4.67	4.38	4.08–4.68	4.29	3.98–4.60	3.43	3.12–3.74	2.9	2.58–3.22

*CI* confidence interval, *Out* outer, *In* inner, *SF* ETDRS subfield, *Nasal periphery* test-points nasal to the outer nasal SF, *Temporal periphery* test-points temporal to the outer temporal SF.

As an alternative, a combined approach of shortened FCP testing and prediction of sensitivity was probed, given that a brief FCP examination is not very burdensome for patients. Addition of 7 test-points (feature-set 4) and 13 test-points (feature-set 5) decreased the MAE (outer resampling) for the prediction of sensitivity at the remaining loci to 4.11 dB [3.88–4.35] and 3.89 dB [3.67–4.10] (Fig. 2 and Table 2). Again, this was markedly better than the corresponding null model MAE estimate of 6.57 dB [6.08–7.07].

### Comparison of predictions and perimetry test–retest-reliability

For 120 eyes of 92 patients, intra-session test–retest examinations were available. The point-wise mean absolute test–retest difference estimate was 3.51 dB [3.11–3.91] for these patients. This MAE values was slightly lower compared to corresponding feature-set 5 prediction MAE estimate of 3.80 dB [3.56–4.03] for the same subset of patients with intra-session test–retest examinations.

**Figure 3:**
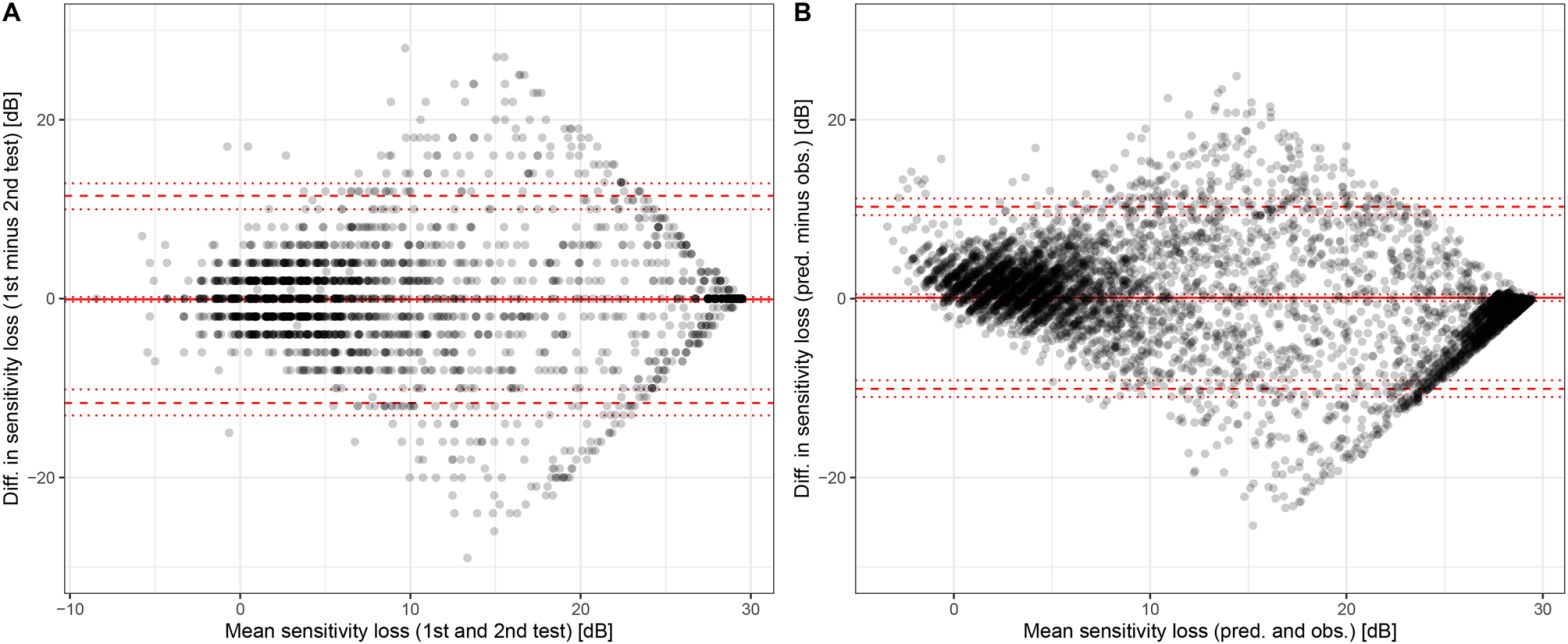
Point-wise retest-variability and prediction accuracy. The differences (diff.) between the first and second microperimetry test were overall slightly larger (**A**) compared to the differences between the cross-validated predictions (pred., feature-set 5) and observations (obs., **B**). Severe degrees of sensitivity loss (compared to normative data) appear to be slightly underestimated by the random forest-based predictions. Please note, for the right plot, twice the number of points were available compared to the left plot, since the random forest predictions were compared to the observations from the first and second test separately. The point-wise differences between single test results to the predictions are larger than the point-wise differences between the average of both test runs and the predictions. Thus, the most conservative estimate of the model performance is shown. The estimates for the mean test–retest difference (solid red line) and the 95% limits of agreement (dashed red lines) were computed with consideration of the hierarchical data structure. The dotted lines indicate the 95% confidence intervals for the test–retest difference and limits of agreement. *dB* decibels.

However, based on the root-mean-squared error (RMSE) estimates, which penalize outliers more than the MAE estimates, the test–retest RSME with 6.04 dB [5.41–6.61] was larger than the prediction RSME of 5.49 dB [5.2–5.77]. Bland–Altman plots comparing the test–retest differences and the prediction-observation differences further reveled no possibly biasing learning effect between first and second FCP test (mean difference around 0). Further the plots underscored that the accuracy of random forest-based predictions using feature-set 5 was indeed comparable to the retest-variability (Fig. 3).

### Importance of imaging biomarkers for retinal sensitivity

**Figure 4:**
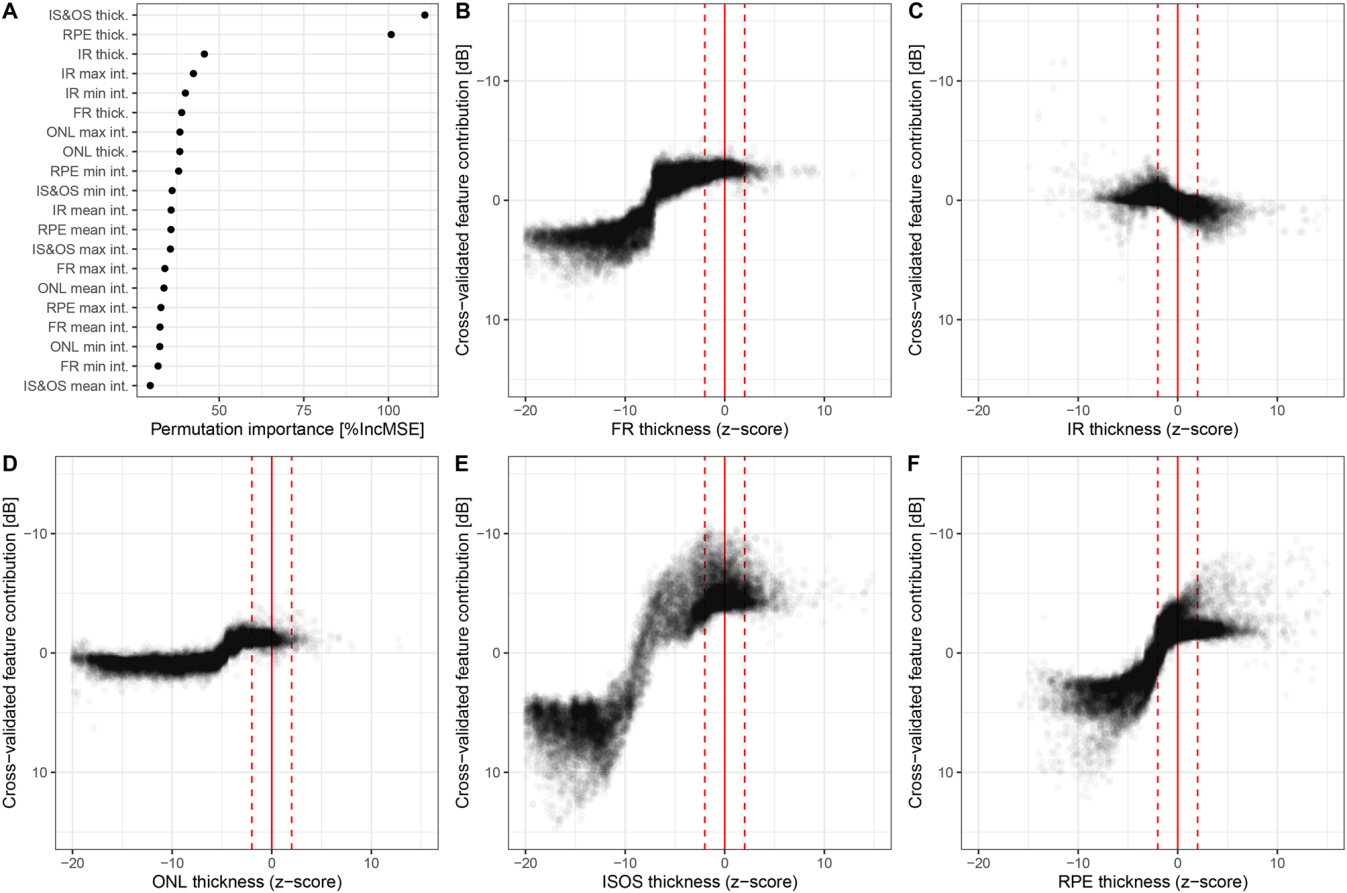
Feature importance. (**A**) The panel shows the permutation feature importance of layer thickness (thick.) as well as mean, maximal (max) and minimal (min) intensity projections (int.) in terms of the percentage of increase in mean squared error (%IncMSE). Overall, the photoreceptor inner and outer segments (IS&OS) thickness and retinal pigment epithelium (RPE) thickness were the two most important features. The permutation feature importance of the other feature sets can be found in Supplementary Fig. S2. (**B**–**F**) The other panels demonstrate the feature contributions [dB] of the respective retinal layer thickness. The dashed lines show the normal range (− 2 to + 2 normative standard deviations) for the thicknesses. The y-axis for these plots was inverted to ensure that sensitivity loss is plotted downwards. The so-called goodness-of-visualization R^2^ metric^25^, which denotes the variance of feature contributions explained by these simple X–Y plots was 0.89 for the full retinal (FR) thickness (**B**), 0.42 for the Inner retinal (IR) thickness (**C**), 0.8 for the outer nuclear layer (ONL) thickness (**D**), 0.92 for IS&OS thickness (**E**), and 0.81 for the RPE thickness (**F**).

**Figure 5:**
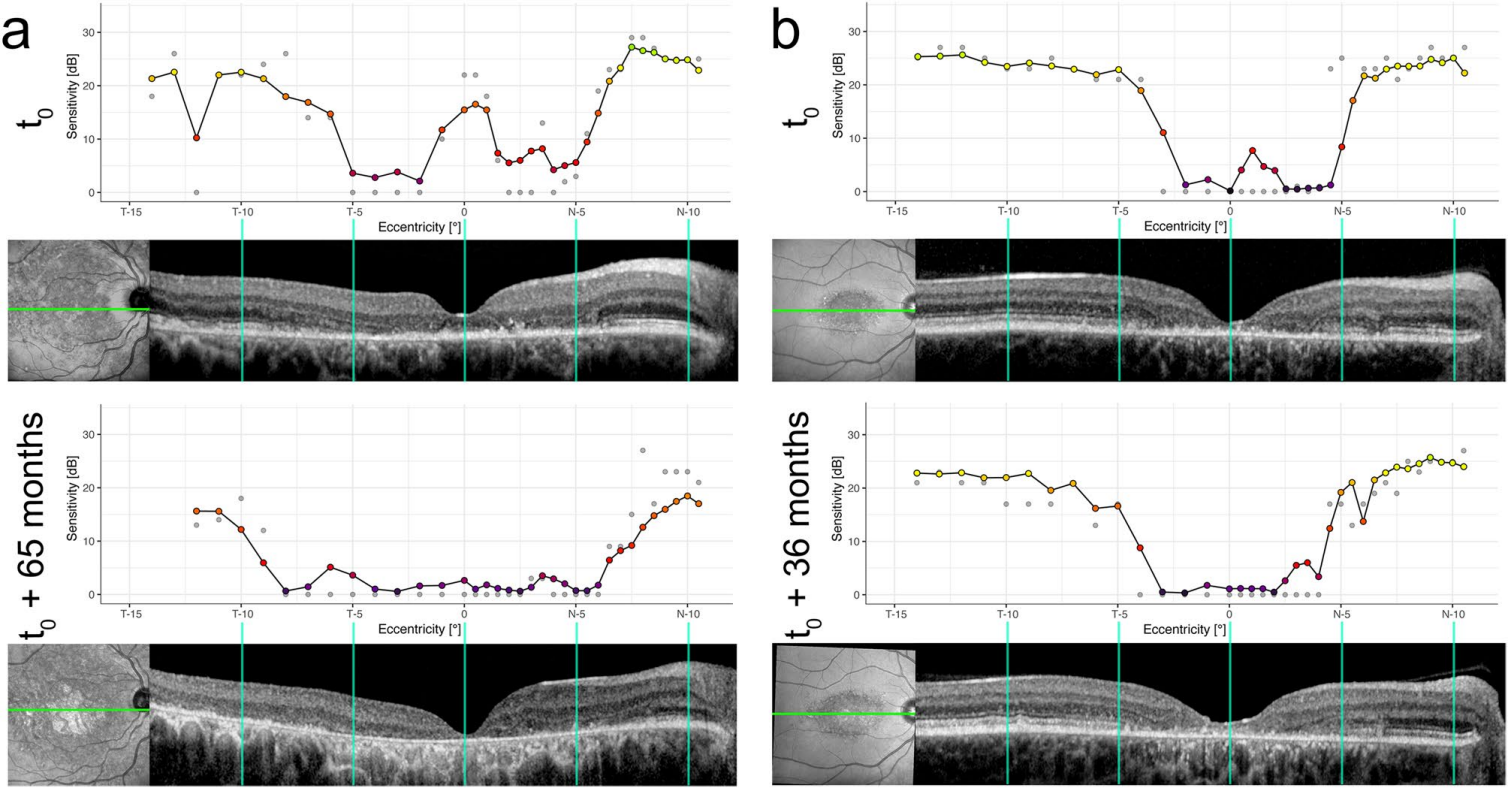
Prediction accuracy of sensitivity-loss over time. The predicted (grey points) and observed (color coded) fundus controled perimetry results (in decibels, dB, top), and corresponding horizontal optical coherence tomography (OCT) B-scan of two exemplary patients are longitudinally demonstrated. The green line on the infrared reflectance image indicates the approximate location of the respective OCT B-scan. (**a**) The functional consequence of the loss of foveal non-involvement over time is adequately recognized by the imaging feature based model. Also the growth of outer retinal atrophy is represented by a loss of observed as well as predicted retinal sensitivity in the first patient. (**b**) The second patient exhibits a more diffuse foveal involving retinal thinning, which is also parallelled by a loss of observed and predicted retinal light sensitivity. In the follow-up visit (bottom row), the development of a small spot of outer retinal atrophy (e.g., at approximately N-7) with loss of retinal sensitivity could correctly be predicted. A summary of the individual longitudinal data can be found in Supplementary Fig. S3.

Analysis of the permutation importance revealed, that the IS&OS thickness (median [IQR]) 110.75% IncMSE [119.90, 101.53] and RPE thickness 100.81% IncMSE [96.68, 106.99] were the most important imaging features for the prediction of retinal sensitivity. Graphical analysis of the imaging feature contributions (feature set 1) emphasized the predictive feature importance of the IS&OS and RPE thickness (Fig. 4). The so-called goodness-of-visualization R^2^ of the IS&OS thickness (0.92) and RPE thickness (0.81) indicates that the residual variance of feature contributions not explained by these simple X–Y plots (due to possible interaction effects) is low. Also for the other feature sets, IS&OS and RPE thickness revealed the highest permutation importance values (Supplementary Fig. S2). Figure 5 shows two exemplary patients that demonstrated the functional relevance of the thinning of these outer retinal layers as well as the high accuracy of the machine learning-based predictions longitudinally.

## Discussion

Since its introduction in 1959, the term machine learning covers different approaches to artificial intelligence, enabling computers to learn without being programmed^26^. In the last decade, machine learning techniques have entered visual science, including analysis in the context of retinal imaging^27^. It has recently been shown to offer great potentials in the detection and classification of pathological features^28^, and in the prediction of retinal function^22,29,30^. Based on these developments, this study investigated the possibility of machine learning algorithms to predict spatially-resolved retinal function in STGD1 based on (1) OCT imaging data, (2) indicators of retest variability, (3) functional along with patients’ demographic measures, and (4) brief FCP testing for the first time. The prediction accuracy of the model was comparable with the retest variability (Fig. 3), while the RPE and IS&OS thicknesses were shown to represent the most important predictive imaging parameters. In accordance with previous studies in AMD^22,23^, we used the term ‘inferred sensitivity’ for this machine learning-based analysis strategy. It may serve as a quasi-functional surrogate marker in future clinical trials.

In the view of emerging therapeutic approaches for STDG1^24^, adequate clinical trial design including the selection of suitable endpoints constitutes a prerequisite for the evaluation of potential benefits. As regulatory agencies have previously stated their preference for functional outcome measures^15^, FCP represents a suitable candidate, as it has a broad measurement range, and offers spatially-resolved measures of retinal sensitivity at different predefined retinal locations. Furthermore, retinal sensitivity measured by FCP tends to decrease over time in a rather monotonous manner (excluding retest-variability, Supplementary Fig. S3)^31,32^. In contrast, BCVA may frequently plateau in patients (e.g., due to foveal sparing). The disadvantages of FCP include the need for specific microperimetry devices, the duration of the examination, and the dependency on patients’ performance^19^. Accordingly, the demand for slender study designs and the problem of subject fatigue limit the accuracy and usability of FCP. In this context, quasi-functional surrogate markers obtained through machine learning algorithms offer a practicable alternative.

As we demonstrated herein, it is possible to predict FCP results through routine imaging, functional and demographic parameters with a high accuracy validating ‘inferred sensitivity’ as a possible candidate for a future quasi-functional surrogate marker for STGD1. Due to the dependence on 3-dimensional OCT data, the main advantages of ‘inferred sensitivity’ comprise that (1) it could be obtained within a short time frame even in patient unfit for psychophysical testing, (2) it is ubiquitously available, (3) it can theoretically provide a much higher lateral resolution compared to current functional testing (i.e., modern OCT devices have a spatial *en-face* resolution of up to 5.7 µm/pixel), (4) it can represent opposing effect (e.g. reduction of subretinal deposits versus RPE atrophy), which would be inadequately represented by often used endpoints such as central retinal thickness, (5) it could be compared across different retinal diseases, and (6) is susceptible to early changes in disease progression before the development of atrophy. The use of ‘inferred sensitivity’ thereby does not only offer an easily available, accurate and highly susceptible quasi-functional surrogate marker, but also gives an additional diagnostic dimension and allows to enroll patients before the development of RPE atrophy, which is mostly used in trials for STDG1 but thought as the end-stage after a possible point of no return^9,14,33^.

Previous studies revealed distinct structure–function correlations between retinal sensitivity and multimodal imaging in STGD1, albeit with only a limited number of narrowly selected predictors and/or application of linear models^9,34–40^. By using a wide array of potentially predictive variables and machine learning, we could provide more evidence that the association between structure and function in STGD1 is tight. By electing to use a supervised machine learning random forest regression, we could evaluate the feature importance and feature contributions. The fact that the IS&OS and RPE thickness, which represent the anatomical site of phototransduction and photopigment recycling, were the most important features, underscores the biological plausibility of our model. In contrast, in a recent AMD study using a similar methodology, ONL was identified as the most predictive factor^22^. While this difference could be governed by true biological effects between these diseases, it may likely be explained by ‘feature noise’. In the context of AMD and reticular pseudodrusen, precise delineation of the photoreceptor inner and outer segments is challenging, which may have led to a relatively higher importance of the ONL thickness. In contrast, in this cohort of STGD1 patients, the delineation of IS&OS was very much feasible.

The smaller benefit (in comparison to AMD)^22,23^ of adding patient- and eye-specific data to the training-set may be linked to the overall more homogeneous patient cohort in STGD1. In contrast to previous work in AMD^22^, eye characteristics not reflected by OCT imaging (e.g., lenticular opacification) are much less likely to play a major role given the age of the patients. Further, the overall training-set was much larger than in the previous study^22^. Accordingly, the training set may be more or less fully representative of the relationship between retinal function and retinal structure in the STGD1.

As established^41^, the “evidence for surrogacy depends upon (1) the biological plausibility of the relationship, (2) the demonstration in epidemiologic studies of the prognostic value of the surrogate for the clinical outcome and (3) evidence from clinical trials that treatment effects on the surrogate correspond to effects on the clinical outcome”. As stated above, the biological plausibility could be provided for ‘inferred sensitivity’ in STGD1. In contrast to traditional morphologic endpoints that do not or only indirectly represent function, ‘inferred sensitivity’ is quasi-functional itself. Therefore, the second criterion is not fully applicable. Concerning the third criterion, the longitudinal accuracy of the models could be confirmed based on the subset of data with more than one visit in terms of natural-history (Fig. 5). However, models are strictly limited by their applicability domain. For treatment trials, a two-track approach with imaging and (limited) FCP testing appears warranted, since treatment could putatively lead to a structure–function dissociation (e.g., in the case of toxic optic neuropathy).

In principle, a deep-learning approach, as previously suggested in the setting of Macular telangiectasia (MacTel) type 2^42^, to estimate sensitivity directly from SD-OCT images may provide slightly higher prediction accuracies. However, deep-learning models are unsuitable to quantitatively evaluate feature importance, and may produce predictions outside of the outcome range. Notably, towards lower values, our model provided much less biased estimates (Fig. 3) compared to the previous deep-learning approach, which systematically overestimated light sensitivity for locations with reduced sensitivity. Given the high-stakes setting of medicine, it may be preferable to have a two-step pipeline as demonstrated here: first image segmentation (which may be automated using deep-learning), followed by a more parsimonious machine learning model to ensure predictions within the outcome range and examine biological plausibility^22,43^. Somewhat similar, this separation of segmentation/preprocessing and the actual classification has been previously proposed for screening and classification of retinal disease^43^. Of note, the models have been trained in a disease-specific manner and may therefore not be easily applied to other disease entities. However, the modeling pipeline could easily be extended to feature these. While the prediction accuracies should theoretically be similar for mesopic measurements across devices (with adjustment of the dB scale), this may empirically not apply. It has been previously established that the inter-device is suboptimal, which may be (partially) attributed to device-specific floor and ceiling effects^44^. Strengths of this study are the systematic comparison of five feature-sets, differential analysis of the importance of retinal layers on the sensitivity prediction as well as the exploration of longitudinal test–retest data. As the innovative diagnostic tool of quantitative autofluorescence is thought to reveal disease-associated alterations before other changes can be detected^45–47^, the implementation into the machine learning algorithm might be warranted in the future.

In summary, this study investigated a machine learning model to predict spatially-resolved retinal function based on easily available patient data and provided evidence of the high accuracy of this approach in STGD1. IS&OS and RPE thickness were the most predictive imaging parameters. The findings of this study indicate that the use of ‘inferred sensitivity’ as a quasi-functional outcome measure offers the possibility for a refined investigation of possible treatment effects in upcoming interventional trials for STGD1 particularly superior to other functional outcome measures. In the future, this approach may also be expanded for high-resolution mapping of spatially-resolved functional impairment in other retinal dystrophies.

## Methods

### Subjects

Patients with STGD1 were recruited from a clinic dedicated to rare retinal diseases. The diagnosis was based on at least one disease-causing mutation in *ABCA4* (NM_000350.2) and a phenotype consistent with STGD1 including RPE atrophy and flecks^48^. Additional retinal pathology, previous vitreoretinal surgery, or other ocular comorbidities substantially affecting visual function (e.g. relevant media opacity like lenticular changes, amblyopia or optic nerve disease) led to exclusion from the study. Follow-up visits were scheduled at the discretion of the physician and patient. Healthy subjects without retinal pathology or prior ocular surgery served as controls. They were recruited from accompanying persons, students, friends, and colleagues. All subjects underwent a comprehensive ophthalmologic examination including BCVA testing using Early Treatment Diabetic Retinopathy Study (ETDRS) charts, slit lamp examination, indirect ophthalmoscopy, and an imaging protocol after pupil dilation using 0.5% tropicamide and 2.5% phenylephrine. Due to unwished bilateral pupil dilatation and/or restricted time quota, only one eye underwent imaging and functional testing in 17 healthy controls.The study protocol was in accordance with the relevant guidelines and regulations and approved by the Institutional Review Board of the University of Bonn (ethics approval ID: 316/11 and 288/17). Written informed consent conforming to the tenets of the Declaration of Helsinki was acquired from all participants.

### Imaging and functional testing

The standardized retinal imaging protocol consisted of fundus photography (Visucam, Carl Zeiss Meditec, Jena, Germany), AF-imaging (Spectralis HRA, Heidelberg Engineering, Heidelberg, Germany), and spectral domain OCT (Spectralis HRA-OCT, Heidelberg Engineering) capturing volume scans (25° × 30°, 61 scans) with at least 20 frames per scan averaged. Furthermore, patients underwent full-field ERG (Toennies Multiliner Vision 1.70, Hochberg, Germany) testing. Mesopic (i.e., combined cone- and rod-photoreceptor function) FCP was performed using the MAIA device (CenterVue, Padua, Italy), which has an inbuilt confocal scanning laser ophthalmoscope (830 nm, 36.5° × 36.5°, 25 frames per second) that enables automated real-time fundus tracking. The custom-made test pattern consisted of 50 test-points centered on the fovea (based on prior OCT images) and primarily along the horizontal meridian (Fig. 1, modified from the foveo-papillary profile proposed by Cideciyan et al. to cover nasal and temporal macula)^49^, as it represents the whole range of individual disease stages and respective functional impairment independent from the disease severity. The protocol has been described before^31,50^. Briefly, after 20 min of adaptation to the white test background luminance at 1.27 cd/m^2^, retinal sensitivity was obtained using achromatic (400–800 nm) Goldmann III stimuli (duration of 200 ms) and a 4–2 staircase strategy with a dynamic range of 3.6 log units (0.08–318.5 cd/m^2^). One full FCP test was performed before examination was executed to reduce learning effects.

### Disease classification

As potential predictive features (apart for imaging features), we also evaluated conventional disease classifications for STGD1. Patients were classified based on (a) age-of onset into early-onset (≤ 10 years), intermediate-onset (10 < age < 45 years) and late-onset (≥ 45 years)^14^, (b) foveal status into foveal involving and foveal sparing RPE atrophy based on multimodal imaging consisting of OCT and fundus autofluorescence, as well as (c) full-field ERG according to Lois and colleagues^51^: Group 1 included eyes with normal responses on scotopic and photopic full-field ERG, group 2 eyes with normal scotopic responses but reduced (over 2 standard deviations) photopic B-wave and 30-Hz flicker amplitudes and group 3 eyes with ERG reductions involving both rod- and cone-driven responses.

### Image processing and analysis

In order to obtain spatially-resolved structural data at the exact location of the individual FCP stimuli, a proprietary approach was implemented as previously described^22^. First, we performed segmentation of volumetric OCT data using the preset software (Spectralis Viewing Module 6.3.2.0, Heidelberg Engineering, Heidelberg, Germany). The segmentation was then reviewed and, if indicated, manually corrected. For layer thickness, we defined the distance between the internal limiting membrane (ILM) and Bruch’s membrane (BrM) as ‘full retina (FR)’. The ‘inner retina (IR)’ encompasses all layers between the ILM and the outer plexiform layer (OPL)-outer nuclear layer (ONL) boundary^52^. The Henle fiber layer (HFL) was counted towards the ‘ONL’^53^. The photoreceptor ‘inner and outer segments (IS&OS)’ ranged from band 1 (external limiting membrane, ELM) to band 3, and ‘RPE’ from band 3 to BrM (Fig. 1a.b)^52^.

Thickness as well as reflectivity maps (min-/mean-/max-intensity projections) for each layer were transferred as a tab-delimited file to ImageJ (U.S. National Institutes of Health, Bethesda, Maryland, USA). The FCP data was then registered to the retinal *en-face* images using the moving least squares (non-linear) method (alpha 1.0, mesh resolution 64, affine transformation) as implemented in ImageJ. At the exact locations of the FCP stimuli (diameter of 0.43°), the mean thickness for each layer as well as the mean value for the minimum, mean and maximum reflectivity intensity projection maps (i.e., *en-face* maps depicting the reflectivity along each A-scan for each layer) were extracted (Fig. 1c). In summary, five thickness values (FR, IR, ONL, IS&OS, and RPE) and fifteen intensity values were measured for each test-point.

### Preprocessing

Patient data were standardized using normal data of included healthy controls to enhance the interpretability of the structure–function analysis. Without this standardization, disease-specific associations would be ‘occluded’ by trivial associations (e.g., the non-standardized inner retinal thickness [essentially as an indicator of eccentricity] would be predictive of retinal function). Sensitivity values (x) were transformed to sensitivity loss by point-wise comparison to the spatially corresponding normative mean ($x_{{{\text{normative}}}}$). Structural features were standardized (z-scores = (${\text{x}} - {\acute{x}}_{{{\text{normative}}}}$)/SD_normative_). The normative mean (${\acute{x}}_{{{\text{normative}}}}$) and standard deviation value (SD_normative_) for each respective variable and each test-point were derived through mixed model linear regression analysis (respective variable as dependent variable, age as independent variable and eye nested in patient as random effects term). The median patient age was applied as reference.

### Predictive modeling

Predictive modeling was performed in R (version 3.6.1), using the library *randomForest* (version 4.6-14)^54^. Random forest regression was elected as learning algorithm based on its favorable bias–variance trade-off, robustness to multicollinearity and inherent ability to uncover interactions among predictors^55^. Sensitivity loss constituted the target variable for all random forest models. In consideration of randomness in resampling and in fitting of random forest models, an outermost loop was implemented to repeat all modeling steps (outer and inner resampling as well as model fitting) using 7 random seeds.

Nested resampling was applied to estimate the accuracy of the models without optimization bias^56^. Specifically, outer resampling was applied (fivefold cross-vaidation with patient-wise splits) to determine the accuracy with nested inner resampling (again fivefold cross-vaidation with patient-wise splits) to optimze the tuning parameter ‘mtry’. Supplementary Fig. S1 explains graphically the nested-cross validation procedure. The hyperparameter ‘mtry’, which denotes the number of predictors sampled for spitting at each node, was tuned over the values 6, 14, and 22 for the first three feature-sets and over values of 120, 160 and 200 for the last two feature-sets (see. below).

The MAE estimates (i.e., mean of the absolute differences between predicted values and true values) served as measure of goodness-of-fit and were computed in consideration of the data structure (visit nested in eye nested in patient) and averaged across seeds. Five feature-sets with putative predictive variables were compared:

- Feature-set-1 (number of predictors *p* = 20): imaging features only.
- Feature-set-2 (*p* = 22): imaging-features and indicators of retest-variability (false-positive responses, mean reaction time).
- Feature-set 3 (*p* = 26): imaging-features, indicators of retest-variability and further functional and demographic data (fixation stability, BCVA, ERG group, age-of-onset category).
- Feature-set 4 (*p* = 26 + 267 [eye-IDs encoded using a one-hot encoding scheme]): test-results from every 4° (7 test-points) were added for the model fitting (temporal(T)-14°, T-10°, T-6°, T-2°, nasal(N)-2°, N-6°, N-10°) and the eye-IDs were added to allow the model to consider eye-specific characteristics for the predictions.
- Feature-set 5 (*p* = 26 + 267 [eye-IDs encoded using a one-hot encoding scheme]): test-results from every 2° (13 test-points) were added for the model fitting (T-14°, T-12°, T-10°, T-8°, T-6°, T-4°, T-2°, 0°, N-2°, N-4°, N-6°, N-8°, N-10°) and the eye-IDs were added to allow the model to consider eye-specific characteristics for the predictions.

The candidate predictors are described in more detail in Supplementary Table S1.

Further, we provided the MAE estimates for null-models for the feature-sets 1, 2 and 3. These produce the mean sensitivity loss from the respective training-set as “prediction” for the respective test-set. For feature sets 4 and 5, the comparable null-models are based on mean value of the per-eye-specific 13 test-points (cf. above feature set 5), which were then applied as “prediction” to the remaining test-points.

The permutation feature importance values in terms of the percentage of increase in mean squared error (%IncMSE) were used to assess the relative importance of the candidate predictors. The median across all random seed and outer resampling folds was used as permutation accuracy estimates. Feature contribution plots were generated to visualize mapping structures of the random forest model^25^.

## Supplementary Information

Supplementary Information.

## Data Availability

The datasets generated during and/or analyzed during the current study are available from the corresponding author on reasonable request.
