# Supplementary material for: Inferred retinal sensitivity in recessive Stargardt disease using machine learning
Source: Sci Rep. 2021 Jan 14;11:1466. doi: 10.1038/s41598-020-80766-4 (PMC7809282; doi:10.1038/s41598-020-80766-4)
Supplement: Supplementary file 1 — Supplementary Information. [file 41598_2020_80766_MOESM1_ESM.pdf]

Inferred retinal sensitivity in recessive Stargardt disease using machine learning

- Supplementary Information -

Philipp L. Müller[1,2,3,4,*], Alexandru Odainic[1], Tim Treis[5], Philipp Herrmann[1,2], Adnan Tufail[3,4], Frank G. Holz[1,2], Maximilian Pfau[1,6]

[1] *Department of Ophthalmology, University of Bonn, Bonn, Germany*
[2] *Center for Rare Diseases, University of Bonn, Bonn, Germany*
[3] *Moorfields Eye Hospital NHS Foundation Trust, London, UK*
[4] *Institute of Ophthalmology, University College London, London, UK*
[5] *BioQuant, University of Heidelberg, Heidelberg, Germany*
[5] *Department of Biomedical Data Science, Stanford University, Stanford, USA*

Content:

Supplementary Figure S1
Supplementary Figure S2
Supplementary Table S1

Supplementary Figure S1: Nested cross-validation

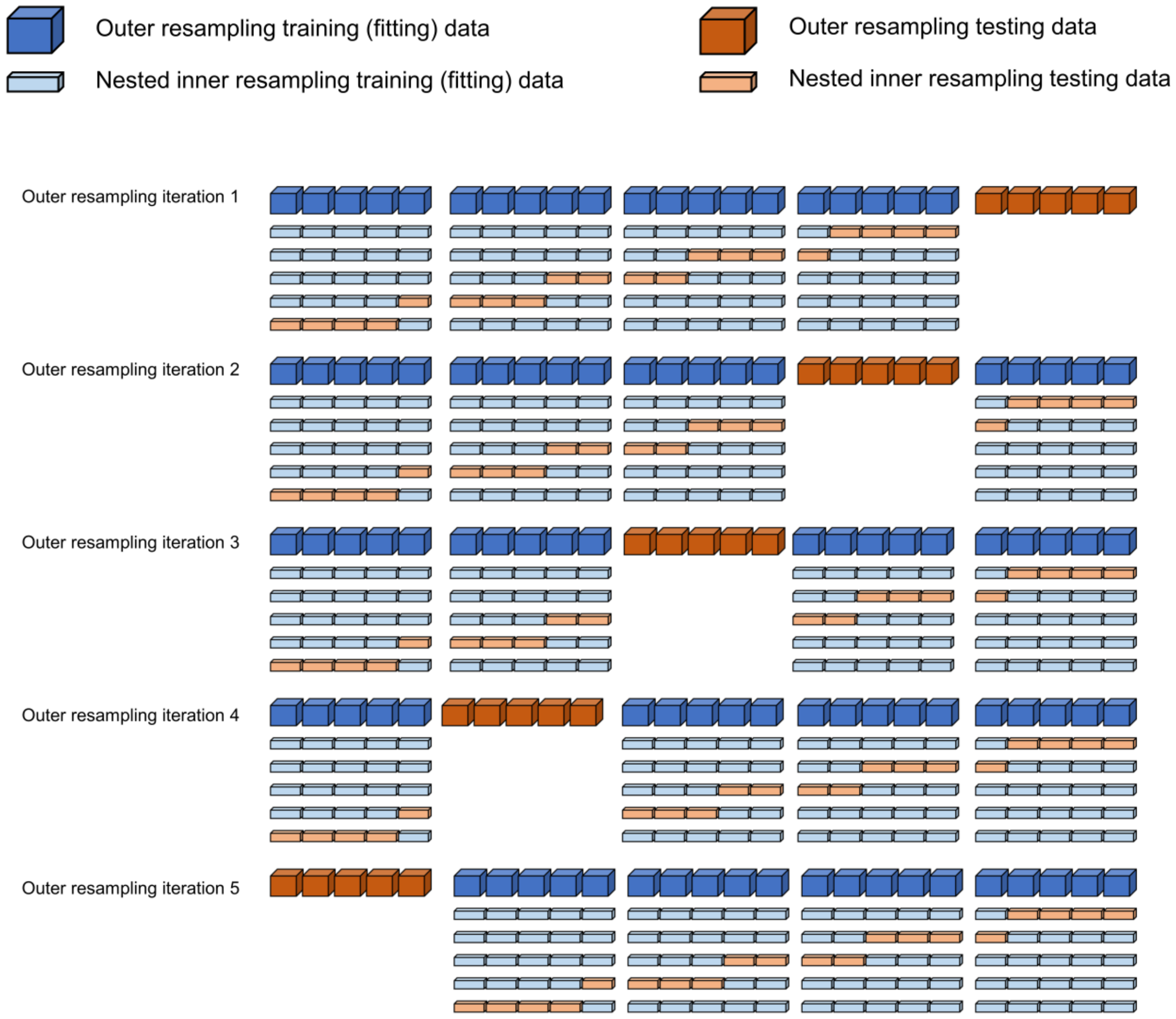

Nested cross-validation was applied to estimate the model accuracy (without optimization bias), while simultaneously tuning the hyperparameter ‘mtry’. Specifically, outer 5-fold cross-validation was applied with nested (i.e., nested within the training splits of the outer resampling) inner 5-fold cross-validation. Given that ocular pathology shows high within-patient correlation, all splits were conducted in a patient-wise manner. To account for the (slight) variability due to the 80/20 splitting of the data in the context of 5-fold cross-validation, all analyses were repeated across seven (1-7) random seeds.

Supplementary Figure S2: Feature importance of further feature-sets

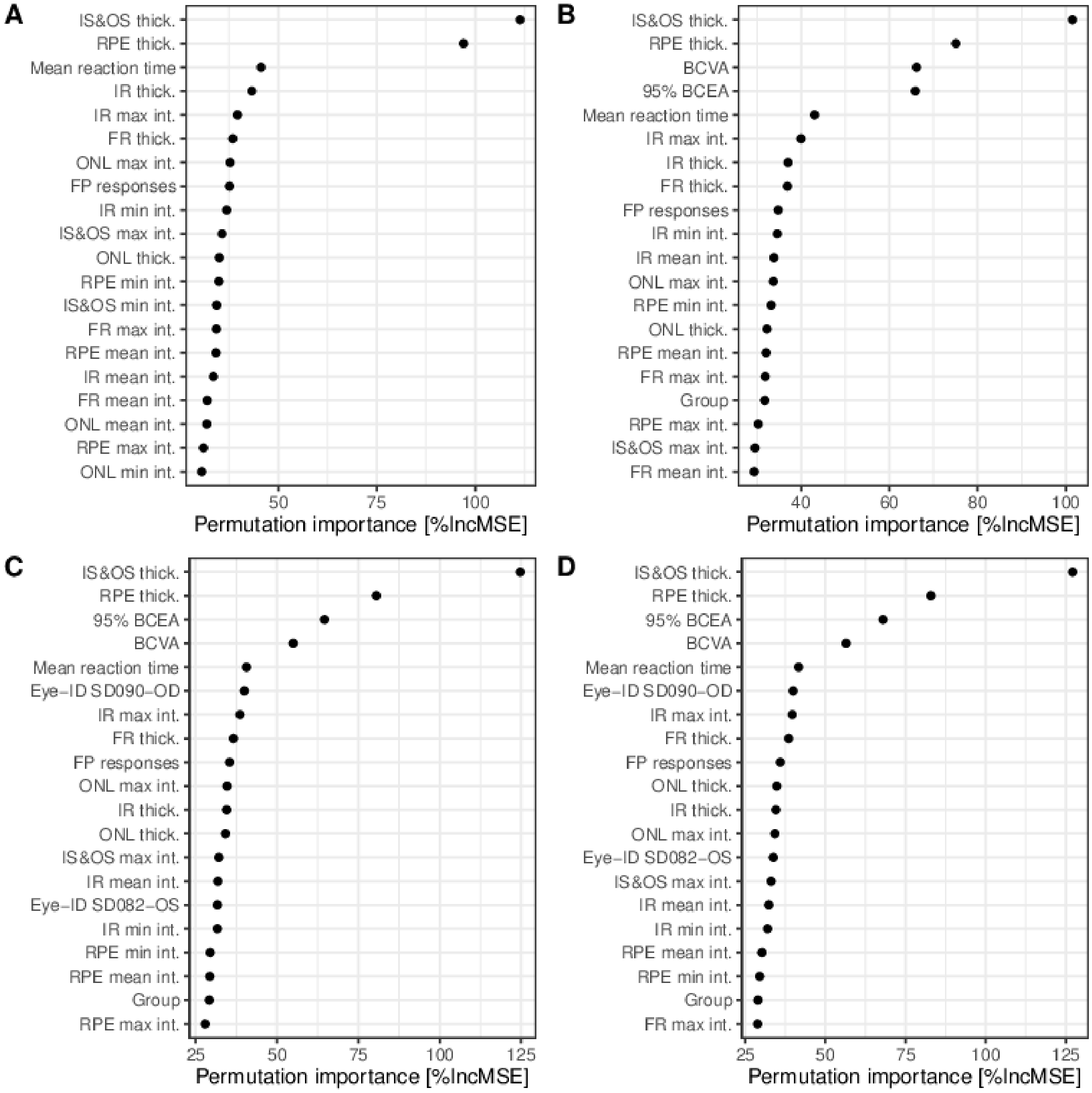

The permutation feature importance in terms of the percentage of increase in mean squared error (%IncMSE) for feature-set 2 (**A**), feature-set 3 (**B**), feature-set 4 (**C**) and feature-set 5 (**D**). The somewhat surprisingly high feature importance for the exam duration is linked to the test-strategy of the used fundus-controlled perimetry device with four initial threshold determinations that are used to inform the start of the staircase procedure for other test-points. Both, relatively normal sensitivity as well as severely reduced sensitivity will lead to short examination duration, whereas irregular loss of sensitivity will lead to long examination durations. FP: false-positive, FR: full retina, int.: intensity projection, IR: inner retina, IS&OS: photoreceptor inner and outer segments, max: maximal, min: minimal, ONL: outer nuclear layer, RPE: retinal pigment epithelium, thick.: layer thickness.

Supplementary Figure S3: Longitudinal observed and predicted change in retinal sensitivity

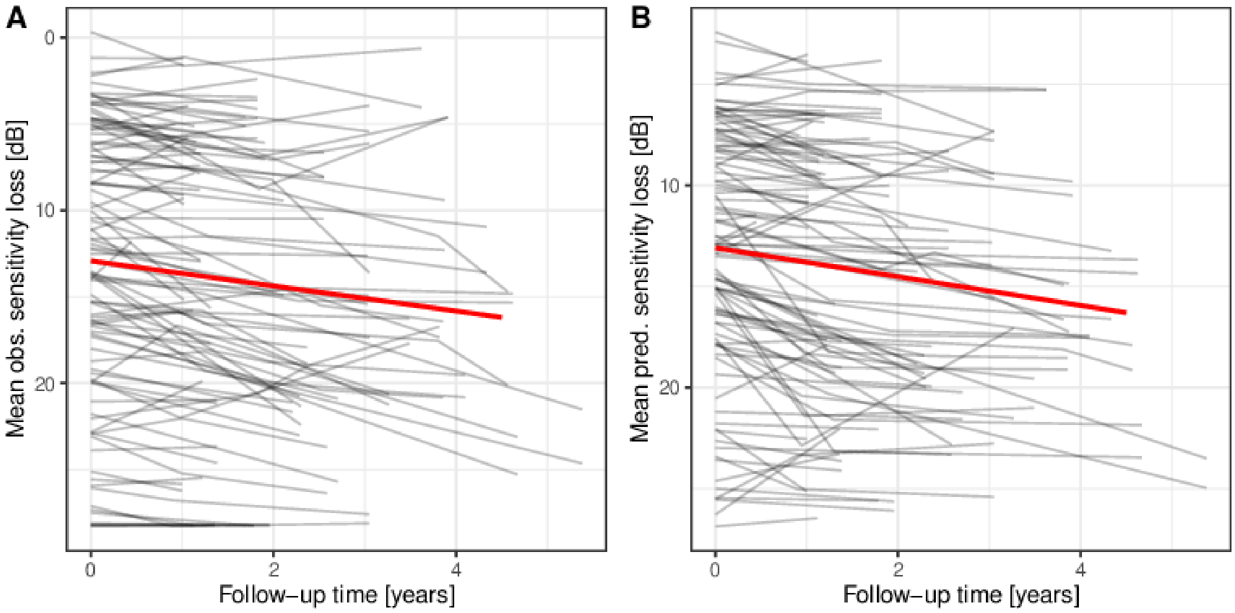

The line-plots show the observed (obs.) change in individual mean retinal sensitivity loss over time (panel A) and the predicted (pred.) change in individual mean retinal sensitivity loss over time (panel B). The predictions are based on feature-set 1 (i.e., SD-OCT data only). The average changes in observed and predicted retinal sensitivity loss are indicated by the red line. A linear mixed model analysis (with test-point nested in eye nested in patient as random effects term) demonstrated that predicted sensitivity explained (marginal R2) 67.7 % of the variability in observed sensitivity in this longitudinal cohort.

Supplementary Table S1. Definition of predictive features

Feature	Feature type	Feature	Feature description
1	Imaging features	FR thickness	Axial thickness between ILM and BM [Z-score]
2		FR max. intensity	Maximum reflectivity between ILM and BM [Z-score]
3		FR mean intensity	Mean reflectivity between ILM and BM [Z-score]
4		FR min. intensity	Minimum reflectivity between ILM and BM [Z-score]
5		IR thickness	Axial thickness between ILM and OPL/ONL boundary [Z-score]
6		IR max. intensity	Maximum reflectivity between ILM and OPL/ONL boundary [Z-score]
7		IR mean intensity	Mean reflectivity between ILM and OPL/ONL boundary [Z-score]
8		IR min. intensity	Minimum reflectivity between ILM and OPL/ONL boundary [Z-score]
9		ONL thickness	Axial thickness between OPL/ONL boundary and ELM [Z-score]
10		ONL max. intensity	Maximum reflectivity between OPL/ONL boundary and ELM [Z-score]
11		ONL mean intensity	Mean reflectivity between OPL/ONL boundary and ELM [Z-score]
12		ONL min. intensity	Minimum reflectivity between OPL/ONL boundary and ELM [Z-score]
13		IS&OS thickness	Axial thickness between ELM and band 3 [Z-score]
14		IS&OS max. intensity	Maximum reflectivity between ELM and band 3 [Z-score]
15		IS&OS mean intensity	Mean reflectivity between ELM and band 3 [Z-score]
16		IS&OS min. intensity	Minimum reflectivity between ELM and band 3 [Z-score]
17		RPE thickness	Axial thickness between band 3 and BM [Z-score]
18		RPE max. intensity	Maximum reflectivity between band 3 and BM [Z-score]
19		RPE mean intensity	Mean reflectivity between band 3 and BM [Z-score]
20		RPE min. intensity	Minimum reflectivity between band 3 and BM [Z-score]
21	Patient reliability indices	False-positive responses	Total of false-positive responses to catch-trial presented to the optic nerve head (similar to Heijl-Krakau method, but with fundus tracking)
22		Average reaction time	Average reaction time [ms]
23	Functional features	95% BCEA	Fixation stability in terms of the 95% bivariate contour ellipse area (BCEA) [deg^2]
24		Age of onset group	Age of onset subclassified as early-onset (≤ 10 years), intermediate-onset (> 10 – < 45 years) and late-onset (≥ 45 years)
25		ERG group	Full-field ERG classification according to Lois and colleagues
26		BCVA	Best-corrected visual acuity [LogMAR]

BM: Bruch's membrane, Deg: degree, ELM: external limiting membrane, FR: full retina, ILM: internal limiting membrane, IR: inner retina, IS&OS: photoreceptor inner and outer segments, LogMAR: Logarithm of the minimum angle of resolution, ONL: outer nuclear layer, OPL: outer plexiform layer, RPE: retinal pigment epithelium.
